# Altered functional connectivity of brainstem nuclei in new daily persistent headache: Evidence from resting‐state functional magnetic resonance imaging

**DOI:** 10.1111/cns.14686

**Published:** 2024-03-22

**Authors:** Wei Wang, Dong Qiu, Yanliang Mei, Xiaoyan Bai, Ziyu Yuan, Xue Zhang, Zhonghua Xiong, Hefei Tang, Peng Zhang, Yaqing Zhang, Xueying Yu, Zhe Wang, Zhaoli Ge, Binbin Sui, Yonggang Wang

**Affiliations:** ^1^ Department of Neurology, Headache Center, Beijing Tiantan Hospital Capital Medical University Beijing China; ^2^ Tiantan Neuroimaging Center of Excellence China National Clinical Research Center for Neurological Diseases Beijing China; ^3^ Department of Radiology, Beijing Neurosurgical Institute, Beijing Tiantan Hospital Capital Medical University Beijing China; ^4^ Department of Neurology The First Affiliated Hospital of Dalian Medical University Dalian Liaoning China; ^5^ Department of Neurology Shenzhen Second People's Hospital Shenzhen Guangdong China

**Keywords:** brainstem, functional connectivity, functional magnetic resonance imaging, new daily persistent headache

## Abstract

**Objectives:**

The new daily persistent headache (NDPH) is a rare primary headache disorder. However, the underlying mechanisms of NDPH remain incompletely understood. This study aims to apply seed‐based analysis to explore the functional connectivity (FC) of brainstem nuclei in patients with NDPH using resting‐state functional magnetic resonance imaging (MRI).

**Methods:**

The FC analysis from the region of interest (ROI) to whole brain voxels was used to investigate 29 patients with NDPH and 37 well‐matched healthy controls (HCs) with 3.0 Tesla MRI. The 76 nuclei in the brainstem atlas were defined as ROIs. Furthermore, we explored the correlations between FC and patients' clinical characteristics and neuropsychological evaluations.

**Results:**

Patients with NDPH exhibited reduced FC in multiple brainstem nuclei compared to HCs (including right inferior medullary reticular formation, right mesencephalic reticular formation, bilateral locus coeruleus, bilateral laterodorsal tegmental nucleus‐central gray of the rhombencephalon, median raphe, left medial parabrachial nucleus, periaqueductal gray, and bilateral ventral tegmental area‐parabrachial pigmented nucleus complex) and increased FC in periaqueductal gray. No significant correlations were found between the FC of these brain regions and clinical characteristics or neuropsychological evaluations after Bonferroni correction (*p >* 0.00016).

**Conclusions:**

Our results demonstrated that patients with NDPH have abnormal FC of brainstem nuclei involved in the perception and regulation of pain and emotions.

## Graphical abstract

This study aims to explore aberrant functional connectivity of brainstem nuclei in patients with NDPH using resting‐state functional magnetic resonance imaging which may provide new insights into the underlying mechanisms of this disease.

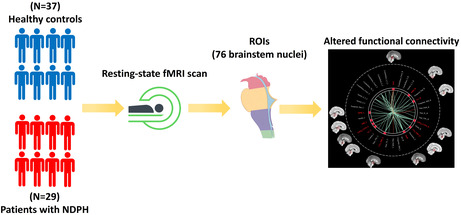

## 1 INTRODUCTION

New daily persistent headache (NDPH) is a rare and under‐recognized primary headache disorder, affecting approximately 0.03 to 0.1% of the general population.^1^ This type of headache is characterized by an acute onset and unremitting headache lasting >3 months according to the diagnostic criteria of *International Classification of Headache Disorders, 3rd Edition* (ICHD‐3).^2^ The pain associated with NDPH lacks distinct characteristics and can manifest as migraine‐like, tension‐type‐like, or exhibit a combination of both features.^2^ This disease is considered highly disabling due to its persistent headache and therapeutic refractoriness. Furthermore, it is often comorbid with sleep disturbances, psychiatric disorders, light‐headedness, concentration problems, sensory disturbances such as numbness or tingling, vertigo, lethargy, and other non‐specific syndromes.^3^, ^4^ Some epidemiological evidence showed that mood disorders were more prevalent in patients with NDPH, and 65.5% of the patients had severe anxiety and 40% had severe depression.^4^ To date, there is no defined pathogenesis and underlying mechanisms of NDPH. Some studies have suggested that NDPH might be associated with viral infection, inflammatory cascade, and the release of pro‐inflammatory cytokines, cervical spine joint hypermobility, etc^1^, ^5^ On balance, it seems that most proposed pathogenesis and underlying mechanisms were somewhat speculative. Thus, NDPH remains enigmatic, and further high‐quality studies are needed for exploring its mechanism.

Neuroimaging studies have provided evidence for exploring the real‐world brain changes in patients with NDPH. Some evidence suggested that there were abnormalities in brain function and structure in adolescents with NDPH.^6^ Our previous studies showed that adult patients with NDPH have unique alterations in hemodynamics, regional homogeneity, multiple frequency amplitudes of low‐frequency fluctuation, cortical activity, and brain functional connectivity (FC) in certain brain regions.^7^, ^8^, ^9^, ^10^ FC is defined as the temporal coincidence of spatially distant neurophysiological events. Over the last decade, an increasing number of studies have applied magnetic resonance imaging (MRI) to explore FC in headache disorders.^11^ With the development of human brain imaging techniques, numerous studies have focused on examining alterations in higher‐order brain regions to understand the central representation of headache and pain. In contrast, fewer investigations have explored the function of brainstem nuclei, primarily due to considerable technical challenges. However, advancements in image analysis techniques, particularly software enabling enhanced spatial normalization of brainstem structures, have significantly improved the accuracy and feasibility of brainstem imaging. The brainstem nucleus plays an important role in central pain pathways related to primary headaches, and some evidence showed that primary headaches were specifically associated with the brainstem nuclei through many neuroimaging studies, which highlight selective brainstem activation.^12^

So far, there were no studies have focused on the FC of brainstem subregions in patients with NDPH. To gain insights into the involvement of specific brainstem nuclei in this rare primary headache disorder, we applied seed‐based resting‐state functional magnetic resonance imaging (rs‐fMRI) analysis to explore FC patterns between region of interest (ROI) to whole brain voxels in patients with NDPH and to explore the correlation between these aberrant FC and clinical variables. We hypothesized that abnormal FC patterns exist in different nuclei of the brainstem in patients with NDPH and that these aberrant FC might be significantly correlated with clinical variables.

## 2 MATERIALS AND METHODS

### 2.1 Study design

This study used a cross‐sectional, case–control design to evaluate FC in patients with NDPH and healthy controls (HCs) from rs‐fMRI data. All participants involved in the registry provided informed consent after receiving comprehensive details about this study. This was a sub‐study of the ongoing China HeadAche DIsorders RegiStry Study (CHAIRS, ClinicalTrials.gov Identifier: NCT05334927). Ethical approval for the study was obtained from the Beijing Tiantan Hospital, Capital Medical University (number: KY2022‐044). This study conforms to the provisions of the Declaration of Helsinki.

### 2.2 Participants

Forty HCs and 31 patients with NDPH were enrolled in the Headache Center, Department of Neurology, Beijing Tiantan Hospital, Capital Medical University from October 2020 to October 2022. This set of data was derived from previously published data.^10^ Patients with NDPH were included if they met the following inclusion criteria: (1) Participants were included if they met the definition of NDPH as per the ICHD‐3 criteria.^2^ This was determined by the following criteria: (A) a persistent headache fulfilling criteria B and C; (B) the headache characterized by a distinct and memorable onset, where the pain becoming persistent and unremitting within 24 h; (C) a headache last for >3 months; and (D) without better explanation by another ICHD‐3 diagnosis; (2) the eligibility of participants for MRI scans (participants without claustrophobic syndrome and metal implant etc.); (3) none of the enrolled patients with NDPH had received prophylactic treatment for a minimum duration of 3 months. Furthermore, the patients diagnosed with NDPH had no prior history of opioid use, hormonal contraceptive use, and excessive utilization of acute therapy; (4) the availability of comprehensive and complete datasets. Patients with NDPH were excluded if they met the following exclusion criteria: (1) before the scan, there were other secondary factors associated with headache; (2) patients in combination with other types of primary headache; (3) pregnancy or lactation, co‐morbidities with other neurological, cardiovascular, cerebrovascular or endocrine system diseases, etc.; (4) incomplete or poor quality of MRI data. The inclusion criteria for HCs were (1) feasibility of MRI scanning (participants without claustrophobic syndrome and metal implant, etc.); (2) no history of neurological or other major systemic diseases; (3) those who well‐matched the age, gender, etc. of patients with NDPH; (4) first‐degree relative without headaches. The exclusion criteria for HCs were (1) pregnancy or lactation; (2) incomplete or poor quality of MRI data.

### 2.3 Demographic data and neuropsychological tests

We collected demographic data, encompassing gender, age, body mass index (BMI), as well as pertinent clinical characteristics of patients diagnosed with NDPH. These characteristics consisted of age at onset, disease duration, pain intensity associated with NDPH, and the related results of neuropsychological tests. We measured the pain intensity of NDPH using the Visual Analog Scale (VAS) and assessed the headache's impact with the Headache Impact Test‐6 (HIT‐6).^13^ Sleep quality and cognitive function were gauged via the Pittsburgh Sleep Quality Index (PSQI) and the Montreal Cognitive Assessment (MoCA), respectively.^14^, ^15^ An overall score above 7 signifies poor sleep quality, while a MoCA score below 26 points to cognitive impairment.^15^, ^16^ Depression and anxiety levels were evaluated using the Patient Health Questionnaire‐9 (PHQ‐9) and the Generalized Anxiety Disorder‐7 (GAD‐7), respectively, with a score of 10 indicating symptoms for both measures.^17^, ^18^

### 2.4 Imaging protocols

We conducted the MRI using a 3.0 Tesla MR scanner (Signa Premier, GE Healthcare, Chicago, IL, USA) equipped with a 48‐channel head coil at the National Neurological Center of Beijing Tiantan Hospital. Participants were instructed to minimize head and neck movements, stay awake, relaxed, and keep their eyes closed throughout the scan. Earplugs and foam padding were used to decrease scanner noise and limit head movement. We obtained T1‐weighted volumetric images using an MP‐RAGE sequence, boasting a 1‐mm isotropic resolution (sagittal acquisition, field of view (FOV) of 256 mm, acquisition matrix of 256, 192 slices, flip angle of 8°, preparation time of 880 ms, recovery time of 400 ms, acceleration factor of 2, acquisition time of 4:00). Resting‐state functional MRI (rs‐fMRI) data were recorded via a multi‐band blood‐oxygenation‐level‐dependent (BOLD) sequence, providing a 2.4‐mm isotropic resolution (transverse acquisition, FOV of 208 mm, acquisition matrix of 86, 65 slices with a multi‐band factor of 6, flip angle of 64°, echo time/repetition time of 39/1000 ms, no in‐plane acceleration or slice gap, and 330 brain volumes).

### 2.5 Data processing and analyses

All MRI data were processed in MATLAB 2016b utilizing both Statistical Parametric Mapping 12 (SPM12) and the Data Processing Assistant for rs‐fMRI Advanced Edition (DPARSFA) (MathWorks, Natick, MA, USA; http://www.rfmri.org/DPARSF). We adhered to the following data preprocessing steps: Firstly, we eliminated the initial 20 time points from each functional time course to mitigate the effect of unstable magnetization. Next, we conducted slice timing, implemented motion correction for headaches, performed spatial normalization, and executed spatial smoothing with a full width at half maximum of 8 mm. These procedures were executed utilizing SPM12 (FIL, London, UK). We excluded participants who exhibited head movements beyond 3 mm or rotation exceeding 2° in any direction. Co‐registered anatomical images were then segmented into white matter, gray matter, and cerebrospinal fluid. These images were subsequently normalized to the standard Montreal Neurological Institute (MNI) space, with a resampled voxel size of 3 × 3 × 3 mm^3^. Finally, we employed DPARSFA for linear trend removal and time bandpass filtering (0.01–0.08 Hz) to diminish the impact of low‐frequency drift and high‐frequency noise.

The FC analysis steps were as follows: Firstly, we identified 76 nuclei in the brainstem atlas as seeds (NITRC: Brainstem Navigator: Tool/Resource Info). Secondly, FC computations of these regions were performed using SPM12. We extracted the time course of the seeds and computed FC between the seed's time course and the average time courses of the whole brain in a voxel‐wise manner, employing Pearson's correlation analysis. Thirdly, to enhance normality, we converted correlation coefficients to *z*‐values using the Fisher *r*‐to‐*z* transformation. Fourthly, we conducted a two‐sample *t*‐test at the voxel level with gender and age as covariates within the brain mask. We set a threshold of *p* < 0.001 (two‐tailed at the voxel level) and used family‐wise error (FWE) correction to adjust to *p* < 0.05 at the cluster level. Lastly, we extracted the average FC values from individual significant clusters for Pearson's correlation analysis with demographic data and neuropsychological tests.

### 2.6 Statistical analyses

The sample size was based on the available data and previous literature.^10^ We described normally distributed data as mean ± SD and non‐normally distributed data as median with interquartile range. The chi‐square test allowed comparison of gender between patients and HCs. To compare normally distributed data across groups, we used the two‐sample t‐test, and for non‐normally distributed data, we applied the Mann–Whitney U test. Pearson's correlation analysis was used to calculate correlations between demographic data, neuropsychological test results, and region of interest (ROI)‐related FC values. We set statistical significance at *p* < 0.05, and all hypothesis tests were two‐tailed. All statistical data were analyzed using SPSS software for Windows (version 25.0; IBM Corp., Armonk, NY, USA). We conducted a two‐sample t‐test at voxel level in SPM12, with gender and age as covariates, to examine FC differences between patients and HCs. For voxel‐based correlation analysis, we used a multiple regression model, considering age and gender as covariates or potential factors; we deemed *p* < 0.05 as significant, applying FWE correction (*p*_FWE_ < 0.05 also considered significant). We implemented multiple corrections when conducting correlation analysis between FC, clinical characteristics, and neuropsychological tests (employing Bonferroni correction, *p* = 0.00016 [0.05/312]).

## 3 RESULTS

### 3.1 Participant profiles and clinical characteristics

We prospectively recruited 31 patients with NDPH and 40 HCs. However, due to incomplete scans and excessive head movements (>3 mm or maximum rotation exceeding 2° in any direction), we excluded 2 patients with NDPH and 3 HCs. Consequently, our study comprised 29 patients with NDPH (15 males, 14 females; age [mean ± SD] 37.00 ± 21.06 years old) and 37 HCs (16 males, 21 females; age [mean ± SD] 34.89 ± 10.96 years old). The demographic details and clinical characteristics of the participants are depicted in Table 1. Importantly, we found no significant differences in demographic information such as age (*p* = 0.627), gender (*p* = 0.497), BMI (*p* = 0.077), and right‐handedness (*p* > 0.999) between the patient and HC groups. Four patients did not have HIT‐6, PHQ‐9, GAD‐7, and PSQI scores and 11 patients did not have MoCA scores due to their reluctance to be assessed by the questionnaires during hospitalization and lost to follow‐up after discharge; thus, we excluded these patients from scale‐associated analysis. Of the patients included, 16 (64.0%) presented severe headache effects (HIT‐6 score ≥ 56), 11 (44.0%) exhibited symptom of depression (PHQ‐9 score ≥ 10), 8 (32.0%) had symptom of anxiety (GAD‐7 score ≥ 10), 16 (64.0%) reported poor quality of sleep (PSQI score >7), and 7 (38.9%) showed cognitive impairment (MoCA score < 26). All participants' structural MRI scans were devoid of any irregularities.

**TABLE 1:** Participants' demographics and clinical characteristics.

	Healthy controls (*n* = 37)	NDPH (*n* = 29)	*p*‐value
Age, years	34.89 ± 10.96	37.00 ± 21.06	0.627
Gender (male/female)	16/21 (43.24/56.76%)	15/14 (51.72/48.28%)	0.497
BMI (kg/m^2^)	22.44 ± 3.04	24.08 ± 4.06	0.077
Right‐handers, *n* (%)	37 (100%)	29 (100%)	> 0.999
Headache laterality, *n* (%)			
Unilateral	NA	4 (13.79%)	NA
Bilateral	NA	25 (86.21%)	NA
Location of headache, *n* (%)			
Frontal region	NA	12 (41.38%)	NA
Temporal region	NA	14 (48.28%)	NA
Parietal region	NA	15 (51.72%)	NA
Occipital region	NA	11 (37.93%)	NA
Periorbital region	NA	1 (3.45%)	NA
Nausea, vomiting, *n* (%)	NA	4 (13.79%)	NA
Photophobia, *n* (%)	NA	11 (37.93%)	NA
Phonophobia, *n* (%)	NA	15 (51.72%)	NA
Age at onset, years	NA	17.00 (13.00, 42.00)	NA
Disease duration, years	NA	3.00 (1.00, 14.00)	NA
VAS score (0–10)	NA	4.00 (3.00, 7.00)	NA
HIT‐6 score (36–78)	NA	64.64 ± 10.94	NA
PHQ‐9 score (0–27)	NA	9.92 ± 6.78	NA
GAD‐7 score (0–21)	NA	6.89 ± 5.17	NA
PSQI score (0–21)	NA	10.20 ± 5.03	NA
MoCA score (0–30)	NA	24.67 ± 4.64	NA

Abbreviations: BMI, body mass index; GAD‐7, Generalized Anxiety Disorder‐7; HIT‐6, Headache Impact Test‐6; MoCA, Montreal Cognitive Assessment; NA, not applicable; NDPH, new daily persistent headache; PHQ‐9, Patient Health Questionnaire‐9; PSQI, Pittsburgh Sleep Quality Index.

### 3.2 The FC of brainstem nuclei between patients and HCs

**FIGURE 1:**
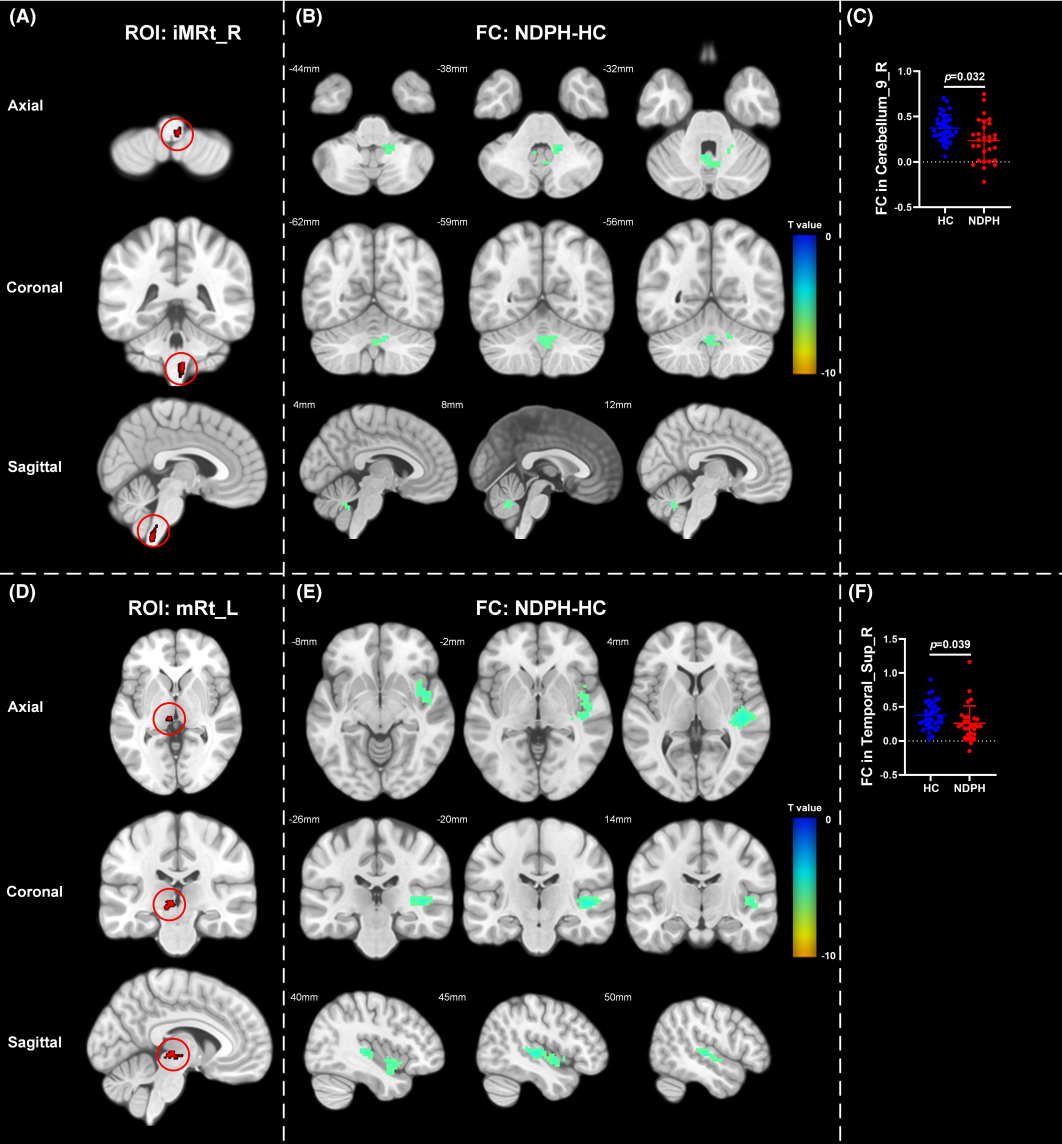
The FC analysis from right iMRt and left mRt to the whole brain voxels in HC and NDPH groups. (A, D) represented the schematic diagram of the ROI (right iMRt and left mRt). (B) Significant FC differences in the right iMRt between HC and NDPH groups. (C) The FC values between the right iMRt and right Cerebellum_9 in HC and NDPH groups. (E) Significant FC differences in the left mRt between HC and NDPH groups. (F) The FC values between the left mRt and right Temporal_Sup in HC and NDPH groups. The color bar shows *t*‐values of the two‐sample *t*‐tests on FC. FC, functional connectivity; HC, healthy control; iMRt, inferior medullary reticular formation; L, left; mRt, mesencephalic reticular formation; NDPH, new daily persistent headache; R, right; ROI, region of interest; Temporal_Sup, superior temporal gyrus.

**FIGURE 2:**
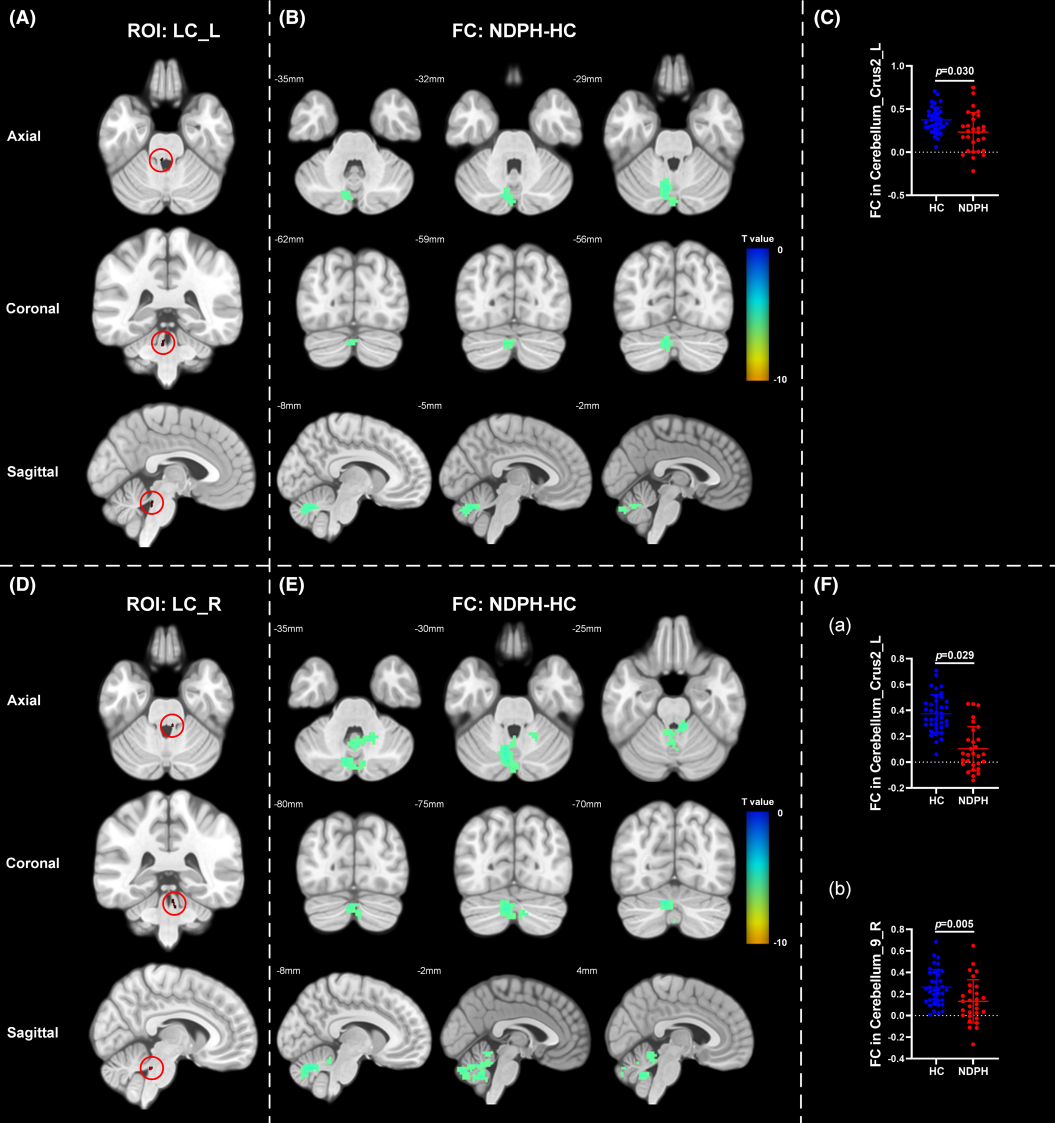
The FC analysis from bilateral LC to the whole brain voxels in HC and NDPH groups. (A, D) represented the schematic diagram of the ROI (bilateral LC). (B) Significant FC differences in the left locus coeruleus between HC and NDPH groups. (C) The FC values between the left LC and left Cerebellum_Crus2 in HC and NDPH groups. (E) Significant FC differences in the right LC between HC and NDPH groups. (F) The FC values between the right LC and left Cerebellum_Crus2, right Cerebellum_9 in HC and NDPH groups. The color bar shows t‐values of the two‐sample *t*‐tests on FC. FC, functional connectivity; HC, healthy control; L, left; LC, locus coeruleus; NDPH, new daily persistent headache; R, right; ROI, region of interest.

**FIGURE 3:**
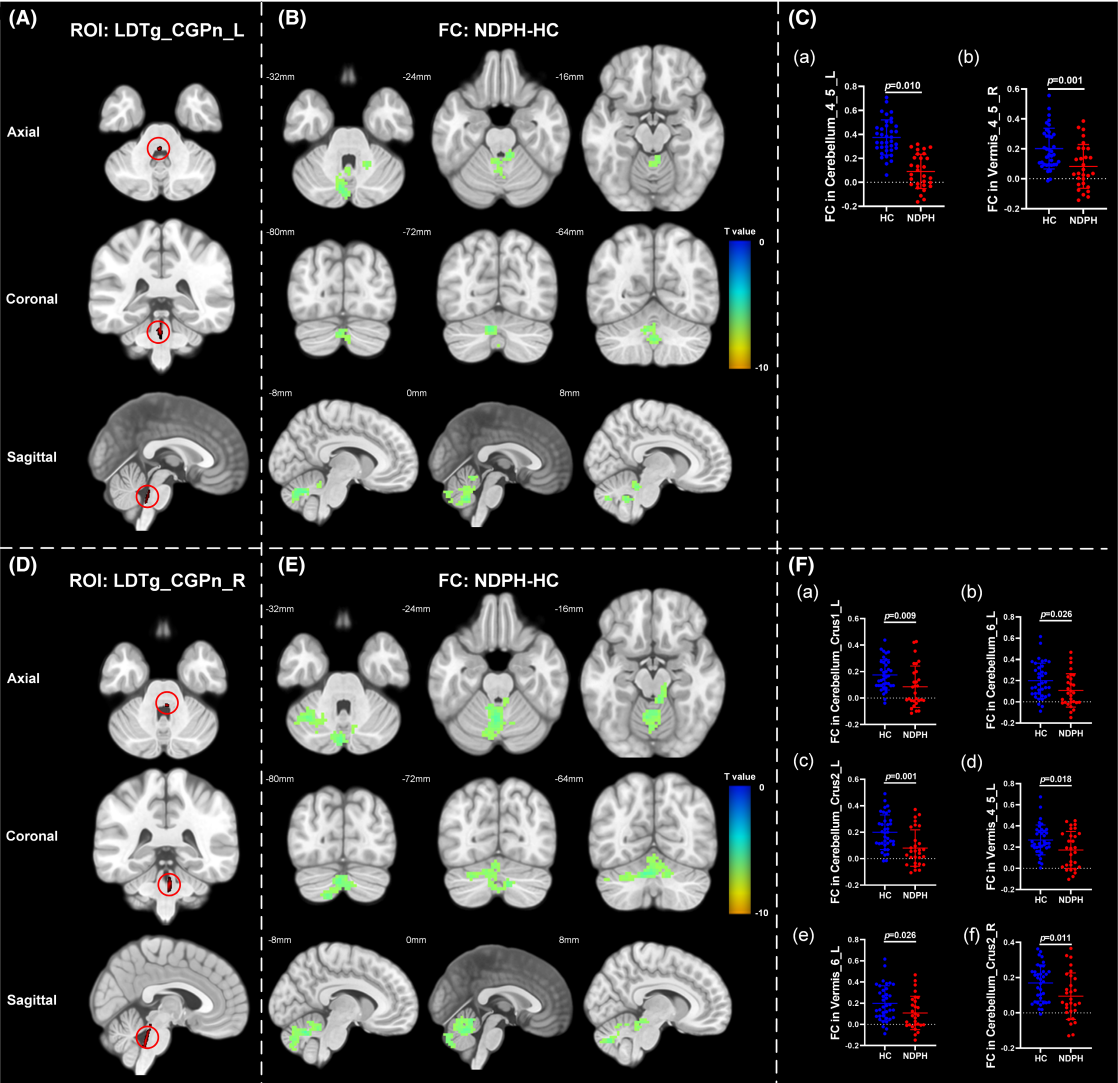
The FC analysis from bilateral LDTg_CGPn to the whole brain voxels in HC and NDPH groups. (A, D) represented the schematic diagram of the ROI (bilateral LDTg_CGPn). (B) Significant FC differences in the left LDTg_CGPn between HC and NDPH groups. (C) The FC values between the left LDTg_CGPn and left Cerebellum_4_5 and right Vermis_4_5 in HC and NDPH groups. (E) Significant FC differences in the right LDTg_CGPn between HC and NDPH groups. (F) The FC values between the right LDTg_CGPn and left Cerebellum_Crus1, bilateral Cerebellum_Crus2 in HC and NDPH groups. The color bar shows *t*‐values of the two‐sample *t*‐tests on FC. FC, functional connectivity; HC, healthy control; L, left; LDTg_CGPn, laterodorsal tegmental nucleus‐central gray of the rhombencephalon; NDPH, new daily persistent headache; R, right; ROI, region of interest.

**FIGURE 4:**
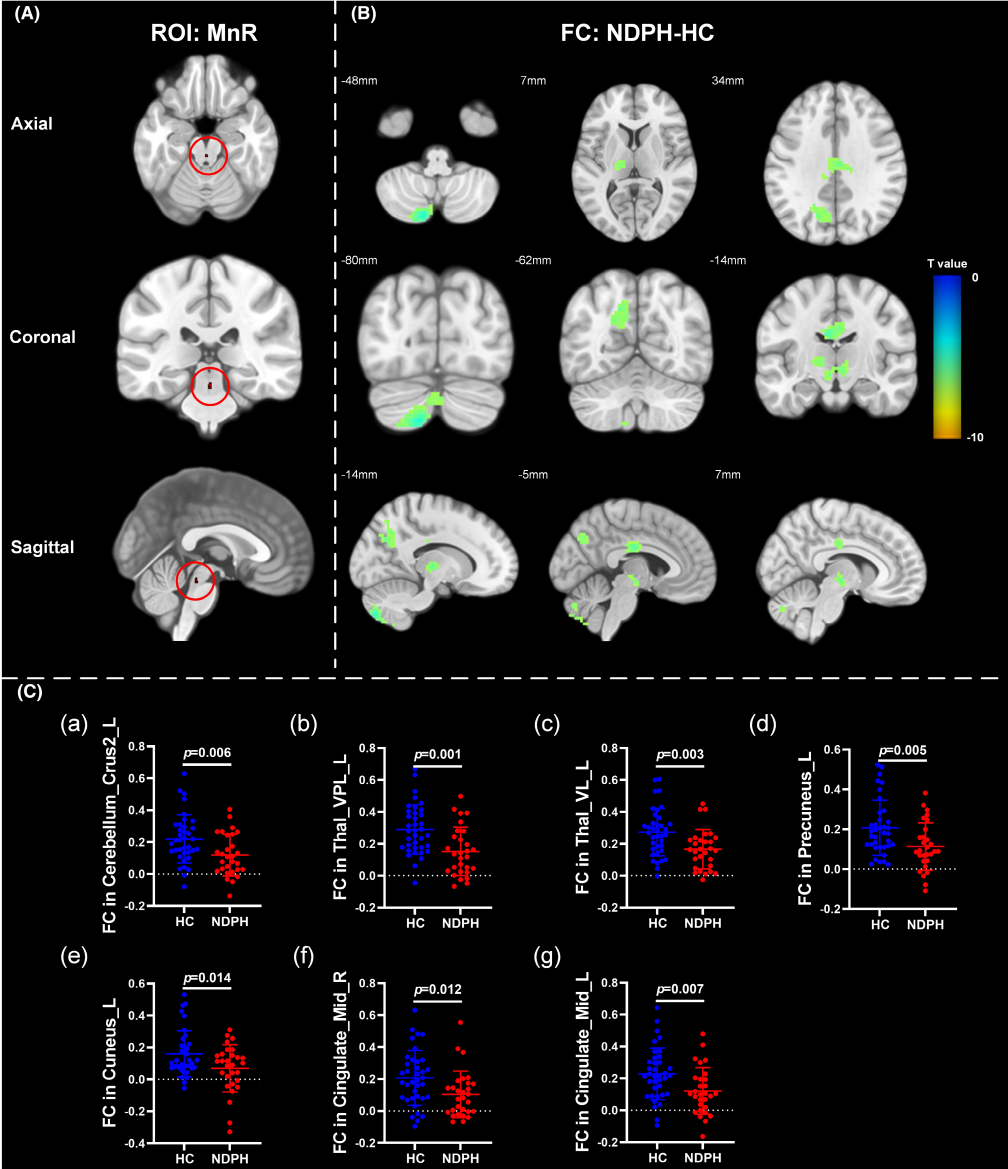
The FC analysis from MnR to the whole brain voxels in HC and NDPH groups. (A) The schematic diagram of the ROI (MnR). (B) Significant FC differences in the MnR between HC and NDPH groups. (C) The FC values between the MnR and left Cerebellum_Crus2, left Thal_VPL, left Thal_VL, left precuneus, left cuneus, and bilateral middle cingulate gyruses in HC and NDPH groups. The color bar shows t‐values of the two‐sample t‐tests on FC. Cingulate_Mid, middle cingulate gyrus; FC, functional connectivity; HC, healthy control; L, left; MnR, median raphe; NDPH, new daily persistent headache; R, right; ROI, region of interest; Thal_VL, ventral lateral thalamus; Thal_VPL, ventral posterolateral thalamus.

**FIGURE 5:**
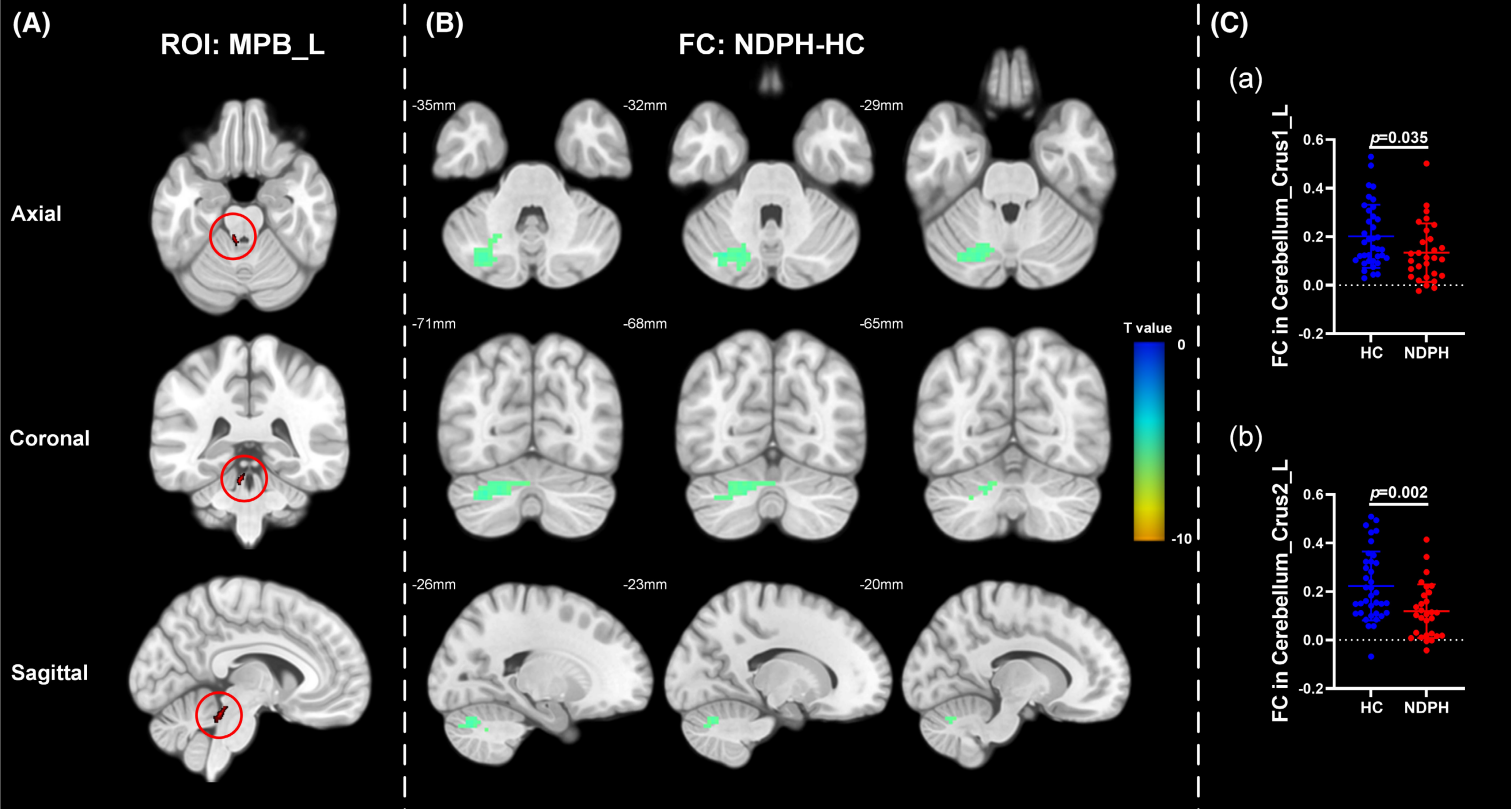
The FC analysis from left MPB nucleus to the whole brain voxels in HC and NDPH groups. (A) The schematic diagram of the ROI (left MPB). (B) Significant FC differences in the left MPB between HC and NDPH groups. (C) The FC values between the left MPB and left Cerebellum_Crus1, left Cerebellum_Crus2 in HC and NDPH groups. The color bar shows *t*‐values of the two‐sample *t*‐tests on FC. FC, functional connectivity; HC, healthy control; L, left; MPB, medial parabrachial nucleus; NDPH, new daily persistent headache; R, right; ROI, region of interest.

**FIGURE 6:**
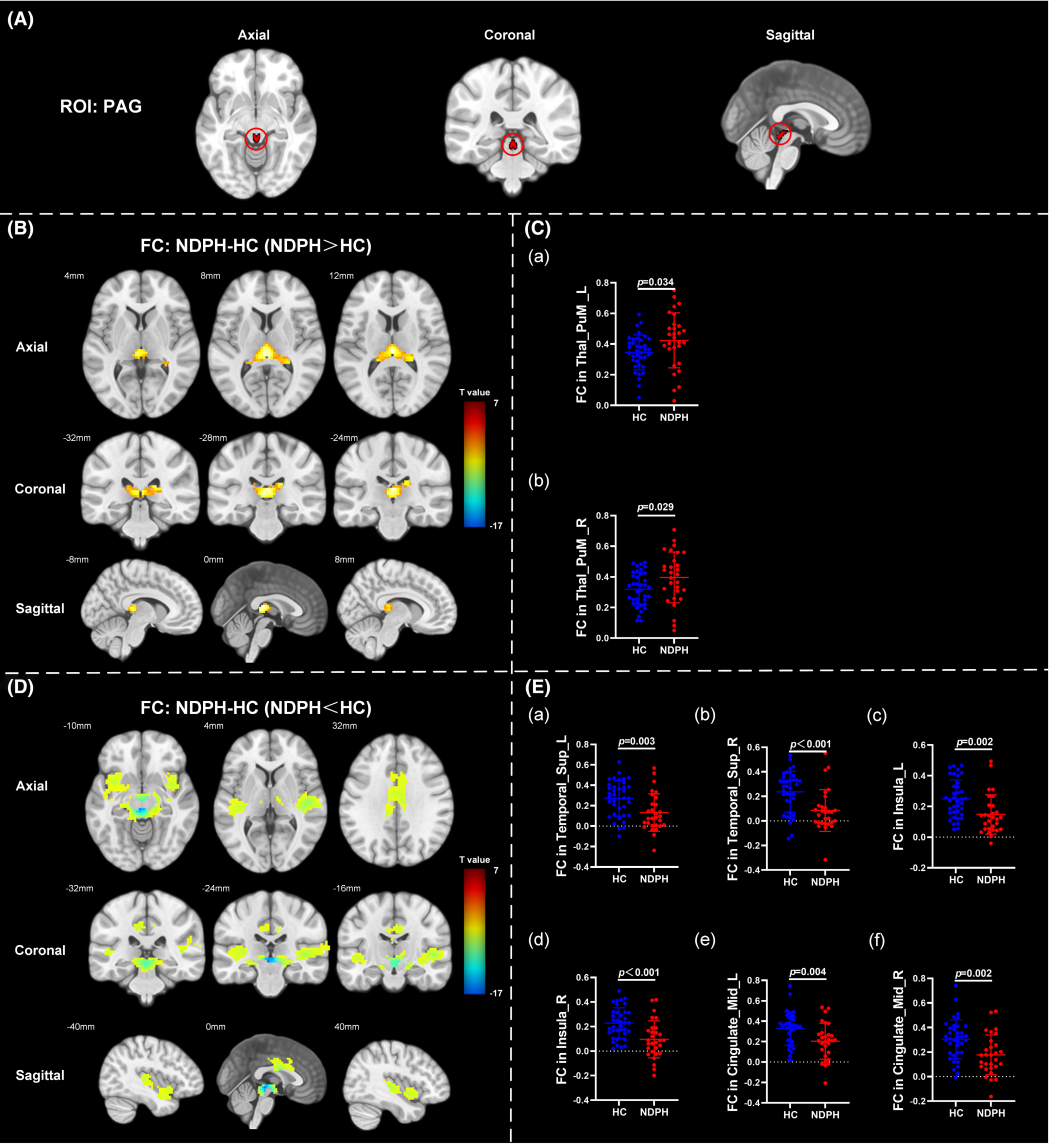
The FC analysis from PAG to the whole brain voxels in HC and NDPH groups. (A) The schematic diagram of the ROI (PAG). (B) Significant increased FC differences in the PAG between HC and NDPH groups. (C) The FC values between the PAG and bilateral Thal_PuM in HC and NDPH groups. (D) Significant decreased FC differences in the PAG between HC and NDPH groups. (E) The FC values between the PAG and bilateral Temporal_Sup, bilateral insula, bilateral Cingulate_Mid in HC and NDPH groups. The color bar shows *t*‐values of the two‐sample t‐tests on FC. Cingulate_Mid, middle cingulate gyrus; FC, functional connectivity; HC, healthy control; L, left; PAG, periaqueductal gray; NDPH, new daily persistent headache; R, right; ROI, region of interest; Temporal_Sup, superior temporal gyrus; Thal_PuM, pulvinar medial thalamus.

**FIGURE 7:**
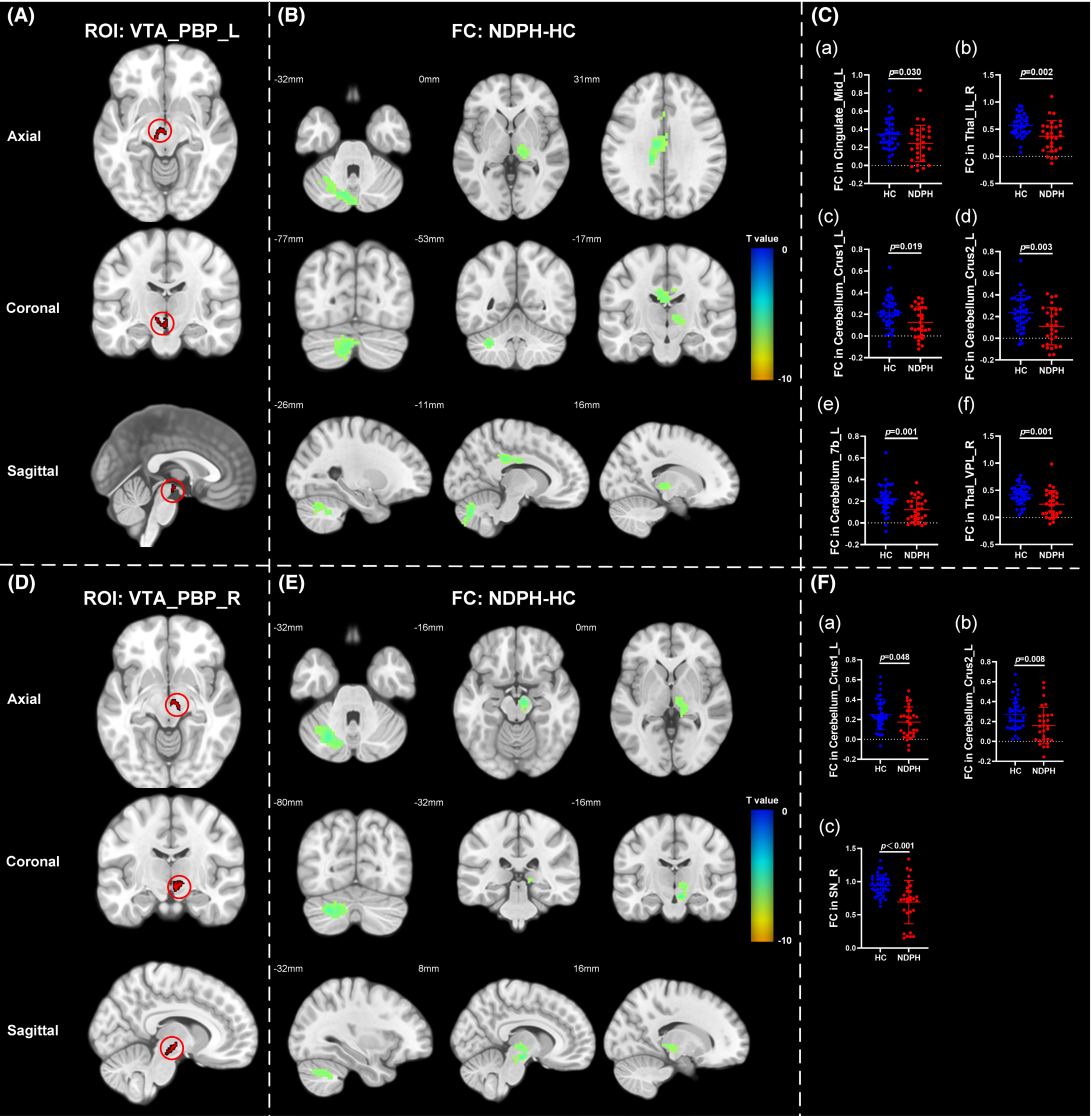
The FC analysis from bilateral VTA_PBP to the whole brain voxels in HC and NDPH groups. (A, D) represented the schematic diagram of the ROI (bilateral VTA_PBP). (B) Significant FC differences in the left VTA_PBP between HC and NDPH groups. (C) The FC values between the left VTA_PBP and left Cingulate_Mid, right Thal_IL, left Cerebellum_Crus1, left Cerebellum_Crus2, left Cerebellum_7b, right Thal_VPL in HC and NDPH groups. (E) Significant FC differences in the right VTA_PBP between HC and NDPH groups. (F) The FC values between the right VTA_PBP and left Cerebellum_Crus1, left Cerebellum_Crus2, right SN in HC and NDPH groups. The color bar shows t‐values of the two‐sample *t*‐tests on FC. Cingulate_Mid, middle cingulate gyrus; FC, functional connectivity; HC, healthy control; L, left; NDPH, new daily persistent headache; R, right; ROI, region of interest; SN, substantia nigra; Thal_IL, intralaminar thalamus; Thal_VPL, ventral posterolateral thalamus; VTA_PBP, Ventral tegmental area‐parabrachial pigmented nucleus complex.

**FIGURE 8:**
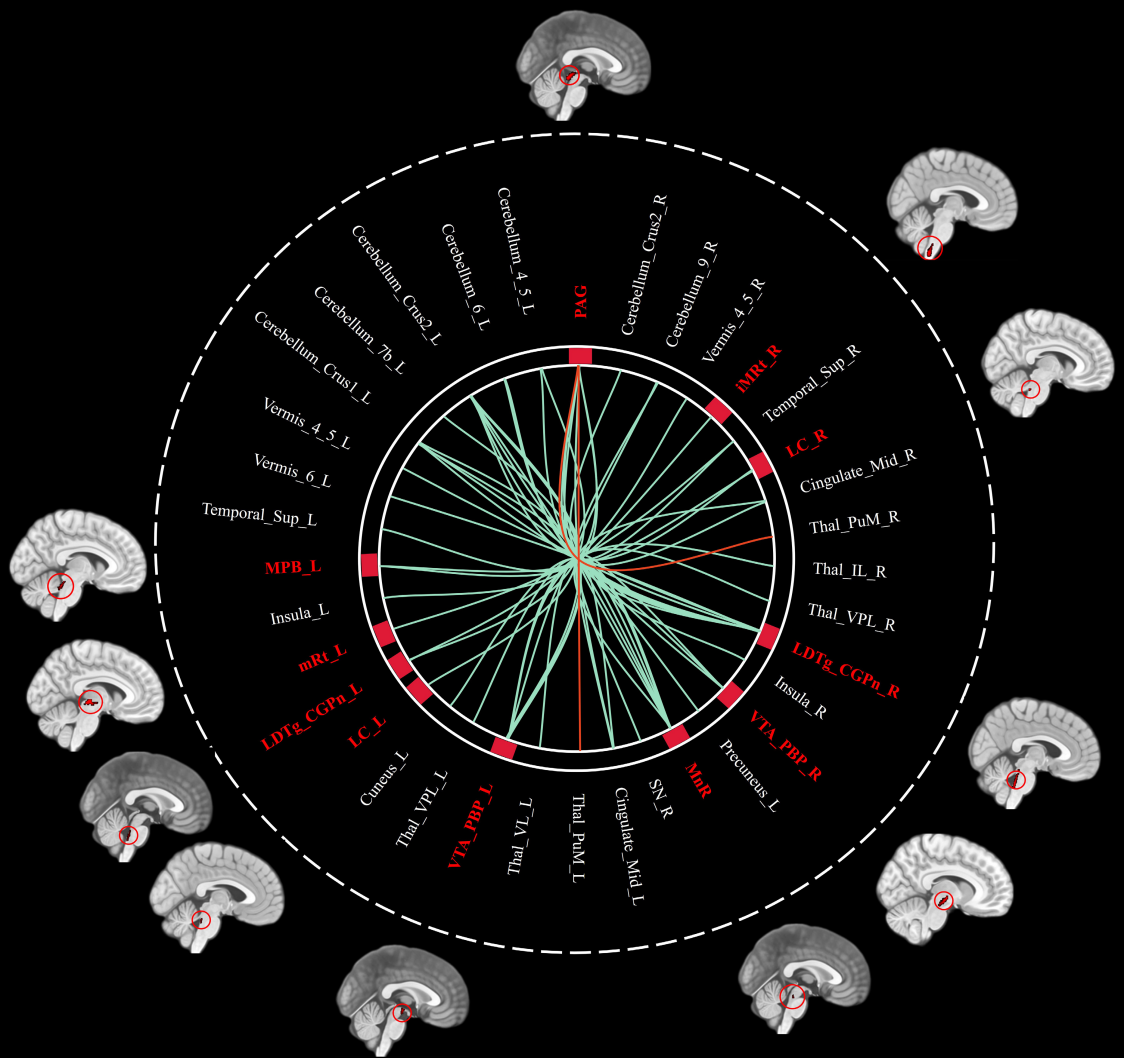
The FC in NDPH vs HC groups. The figure summarizes group differences in seed to the whole brain voxels FC. The region highlights in red represented the region of interests. The green and red curves respectively represented the decreased and increased FC in patients with NDPH. ACC, anterior cingulate cortex; FC, functional connectivity; HC, health control; iMRt, inferior medullary reticular formation; L, left; LC, locus coeruleus; LDTg_CGPn, laterodorsal tegmental nucleus‐central gray of the rhombencephalon; Mid, middle; MnR, median raphe; MPB, medial parabrachial nucleus; mRt, mesencephalic reticular formation; NDPH, new daily persistent headache; PAG, periaqueductal gray; PMnR, paramedian nucleus; R, right; RN, red nucleus; SN, substantia nigra; Sup, superior; Thal_IL, intralaminar thalamus; Thal_PuM, pulvinar medial thalamus; Thal_VL, ventral lateral thalamus; Thal_VPL, ventral posterolateral thalamus; VTA_PBP, ventral tegmental area‐parabrachial pigmented nucleus complex.

Based on 76 nuclei in the brainstem atlas, we finally found abnormal FC in 11 seeds including right inferior medullary reticular formation (iMRt), right mesencephalic reticular formation (mRt), bilateral locus coeruleus (LC), bilateral laterodorsal tegmental nucleus‐central gray of the rhombencephalon (LDTg_CGPn), median raphe (MnR), left medial parabrachial nucleus (MPB), periaqueductal gray (PAG), and bilateral ventral tegmental area‐parabrachial pigmented nucleus complex (VTA_PBP). The results were detailed as follows: (1) The FC between the right iMRt and right Cerebellum_9 (Figure 1A–C; *p* = 0.032) were significantly decreased in patients compared to HCs. (2) The FC between the left mRt and right superior temporal gyrus (Figure 1D–F; *p* = 0.039) were significantly decreased in patients compared to HCs. (3) The FC between the left LC and left Cerebellum_Crus2 (Figure 2A–C; *p* = 0.030) were significantly decreased in patients compared to HCs. (4) The FC between the right LC and left Cerebellum_Crus2 (Figure 2D–F; *p* = 0.029), right Cerebellum_9 (Figure 2D–F; *p* = 0.005) were significantly decreased in patients compared to HCs. (5) The FC between the left LDTg_CGPn and left Cerebellum_4_5 (Figure 3A–C; *p* = 0.010), right Vermis_4_5 (Figure 3A–C; *p* = 0.001) were significantly decreased in patients compared to HCs. (6) The FC between the right LDTg_CGPn and left Cerebellum_Crus1 (Figure 3D–F; *p* = 0.009), left Cerebellum_6 (Figure 3D–F; *p* = 0.026), bilateral Cerebellum_Crus2 (Figure 3D–F; L: *p* = 0.001; R: *p* = 0.011), left Vermis_4_5 (Figure 3D–F; *p* = 0.018), left Vermis_6 (Figure 3D–F; *p* = 0.026) were significantly decreased in patients compared to HCs. (7) The FC between the MnR and left Cerebellum_Crus2 (Figure 4A–C; *p* = 0.006), left ventral posterolateral thalamus (Figure 4A–C; *p* = 0.001), left ventral lateral thalamus (Figure 4A–C; *p* = 0.003), left precuneus (Figure 4A–C; *p* = 0.014), bilateral middle cingulate gyrus (Figure 4A–C; L: *p* = 0.007; R: *p* = 0.012) were significantly decreased in patients compared to HCs. (8) The FC between the left MPB and left Cerebellum_Crus1 (Figure 5A–C; *p* = 0.035), left Cerebellum_Crus2 (Figure 5A–C; *p* = 0.002) were significantly decreased in patients compared to HCs. (9) The FC between the PAG and bilateral pulvinar medial thalamus (Figure 6A–C; L: *p* = 0.034; R: *p* = 0.029) were significantly increased in patients compared to HCs. In contrast, the FC between the PAG and bilateral superior temporal gyrus (Figure 6D,E; L: *p* = 0.003; R: *p* < 0.001), bilateral insula (Figure 6D,E; L: *p* = 0.002; R: *p* < 0.001), bilateral middle cingulate gyrus (Figure 6 D–E; L: *p* = 0.004; R: *p* = 0.002) were significantly decreased in patients compared to HCs. (10) The FC between the left VTA_PBP and left middle cingulate gyrus (Figure 7A–C; *p* = 0.030), right intralaminar thalamus (Figure 7A–C; *p* = 0.002), left Cerebellum_Crus1 (Figure 7A–C; *p* = 0.019), left Cerebellum_Crus2 (Figure 7A–C; *p* = 0.003), left Cerebellum_7b (Figure 7A–C; *p* = 0.001), right ventral posterolateral thalamus (Figure 7A–C; *p* = 0.001) were significantly decreased in patients compared to HCs. (11) The FC between the right VTA_PBP and left Cerebellum_Crus1 (Figure 7D,E; *p* = 0.048), left Cerebellum_Crus2 (Figure 7D–F; *p* = 0.008), right substantia nigra (Figure 7D–F; *p* < 0.001) were significantly decreased in patients compared to HCs. The schematic representation of abnormal FC is depicted in Figure 8. The specific nuclei in the brainstem coordinates and cluster details are listed in Table 2.

**TABLE 2:** Brainstem region with aberrant functional connectivity.

Brain region	Side	MNI coordinates			Peak *t*‐value	Cluster size	Cluster level *p*_FWE corr._
		*x*	*y*	*z*			
**Seed: iMRt_R; FC: patients < HCs**							
*Cluster 1*		15	−48	−42	4.36	85	0.038
Cerebellum_9	R						
**Seed: mRt_L; FC: patients < HCs**							
*Cluster 1*		48	−21	0	5.13	247	0.001
Temporal_Sup	R						
**Seed: LC_L; FC: patients < HCs**							
*Cluster 1*		−9	−66	−30	4.31	81	0.033
Cerebellum_Crus2	L						
**Seed: LC_R; FC: patients < HCs**							
*Cluster 1*		−3	−57	−39	4.53	348	<0.001
Cerebellum_Crus2	L						
Cerebellum_9	R						
**Seed: LDTg_CGPn_L; FC: patients < HCs**							
*Cluster 1*		−3	−54	−12	4.48	188	<0.001
Cerebellum_4_5	L						
Vermis_4_5	R						
**Seed: LDTg_CGPn_R; FC: patients < HCs**							
*Cluster 1*		−3	−20	−27	4.66	630	<0.001
Cerebellum_Crus1	L						
Cerebellum_6	L						
Cerebellum_Crus2	L						
Vermis_4_5	L						
Vermis_6	L						
Cerebellum_Crus2	R						
**Seed: MnR; FC: patients < HCs**							
*Cluster 1*		−12	−81	−48	5.48	162	0.002
Cerebellum_Crus2	L						
*Cluster 2*		−15	−18	3	4.30	100	0.023
Thal_VPL	L						
Thal_VL	L						
*Cluster 3*		−18	−66	30	4.23	122	0.010
Precuneus	L						
Cuneus	L						
*Cluster 4*		−9	−15	27	4.52	118	0.011
Cingulate_Mid	R						
Cingulate_Mid	L						
**Seed: MPB_L; FC: patients < HCs**							
*Cluster 1*		−30	−72	−33	4.29	99	0.023
Cerebellum_Crus1	L						
Cerebellum_Crus2	L						
**Seed: PAG; FC: patients > HCs**							
*Cluster 1*		0	−24	12	6.96	253	0.001
Thal_PuM	L						
Thal_PuM	R						
**Seed: PAG; FC: patients < HCs**							
*Cluster 1*		−3	−27	−6	16.19	998	<0.001
Temporal_Sup	R						
Insula	R						
*Cluster 2*		0	0	24	6.07	431	<0.001
Cingulate_Mid	R						
Cingulate_Mid	L						
*Cluster 3*		−36	9	−9	4.87	454	<0.001
Temporal_Sup	L						
Insula	L						
**Seed: VTA_PBP_L; FC: patients < HCs**							
*Cluster 1*		6	−12	27	4.78	245	<0.001
Cingulate_Mid	L						
Thal_IL	R						
*Cluster 2*		−30	−56	−36	4.44	243	<0.001
Cerebellum_Crus1	L						
Cerebellum_Crus2	L						
Cerebellum_7b	L						
*Cluster 3*		6	−33	−12	4.44	92	0.038
Thal_VPL	R						
**Seed: VTA_PBP_R; FC: patients < HCs**							
*Cluster 1*		−27	−69	−36	5.39	210	0.001
Cerebellum_Crus1	L						
Cerebellum_Crus2	L						
*Cluster 2*		9	−15	−15	4.73	109	0.026
SN	R						

*Note*: Brain region localizations were performed using an automatic anatomical labeling atlas 3rd edition, and the number of voxels of the anatomical regions in which the cluster extends to are reported.

Abbreviations: ACC, anterior cingulate cortex; FC, functional connectivity; FWE corr., family‐wise error correction; HCs, healthy controls; iMRt, inferior medullary reticular formation; L, left; LC, locus coeruleus; LDTg_CGPn, laterodorsal tegmental nucleus‐central gray of the rhombencephalon; Mid, middle; MNI, Montreal Neurological Institute; MnR, median raphe; MPB, medial parabrachial nucleus; mRt, mesencephalic reticular formation; PAG, periaqueductal gray; PMnR, paramedian nucleus; R, right; RN, red nucleus; SN, substantia nigra; Sup, superior; Thal_IL, intralaminar thalamus; Thal_PuM, pulvinar medial thalamus; Thal_VL, ventral lateral thalamus; Thal_VPL, ventral posterolateral thalamus; VTA_PBP, ventral tegmental area‐parabrachial pigmented nucleus complex.

### 3.3 Correlations between FC and patients' clinical variables

In this study, we compared correlations between FC of brainstem nuclei and clinical variables in patients with NDPH. No significant correlation was found between the FC and patients' clinical variables, including the disease duration, headache intensity, the score of HIT‐6, GAD‐7, PHQ‐9, PSQI, MoCA after controlling age and gender (Table S1).

## 4 DISCUSSION

Here, we applied a seed‐based approach to explore resting‐state FC of brainstem and its possible correlation to clinical variables in patients with NDPH. Interestingly, we found that patients had different brain network patterns in multiple subnuclei of brainstem. This provides us with neuroimaging evidence to elucidate the potential mechanism of this rare primary headache.

### 4.1 The locus coeruleus in headache and pain

The LC serves as a prominent origin of noradrenergic projections within the central nervous system. It dispatches extensive and selective projections to various regions, including the neocortex, spinal cord, hypothalamus, hippocampus, cerebellum, and certain parts of the thalamus, thus supplying a significant source of noradrenaline.^12^, ^19^, ^20^ Noradrenaline acts as a powerful modulator of pain, exerting intrinsic control of pain. Besides containing noradrenaline, LC neurons also co‐express other neuropeptides known to be involved in headache are also expressed in the LC, including pituitary adenylate cyclase‐activating peptide, substance P, and neuropeptide Y, etc.^12^, ^21^, ^22^, ^23^, ^24^ Stress and emotional factors are known triggers for both pain and headache, and the LC is a key mediator of the stress response. Stress‐induced activation of the LC and subsequent noradrenaline release can modulate pain sensitivity.^12^, ^25^, ^26^ Current evidence also suggests that the LC has been implicated in the pathophysiology of headache disorders, including migraine and cluster headache.^12^, ^27^ Dysfunctions in LC activity, such as altered firing rates or abnormal release of noradrenaline, may contribute to the initiation and maintenance of headaches.^12^, ^28^ Above all, the locus coeruleus emerges as a critical player in the intricate network of pain and headache modulation. Our results showed that LC has reduced FC to the cerebellum, although there is no direct neural connection between the cerebellum and LC, they can influence each other indirectly through neural networks. For example, the LC regulates the brain's levels of attention and arousal by releasing neurotransmitters such as norepinephrine, and these neurotransmitters may also affect cerebellar activity. This also prompted us to focus on the potential pathogenesis of noradrenaline nuclei in patients with NDPH.

### 4.2 The median raphe in headache and pain

The median raphe is a primary source of serotonergic projections in the central nervous system, and its activation leads to the release of serotonin (5‐HT) throughout the brain.^29^ It has been implicated in the regulation of pain, headache, and emotional states, playing a crucial role in the etiology and treatment of related disorders.^30^ Furthermore, it has been proposed that the serotonin system is involved in the regulation of various behaviors, including aggression, aversive learning, impulsivity, attention, decision‐making, and reward.^29^ Additionally, projections originating from the median raphe nucleus regulate dopaminergic activity in the forebrain. These projections are essential for encoding negative experiences and may have a central role in mood disorders associated with depression and anxiety.^31^, ^32^ NDPH is not only characterized by persistent headache symptoms but often accompanied by psychological symptoms such as depression and anxiety. Our results suggest that these abnormal FC in median raphe may be involved in the pathogenesis of NDPH‐related pain, depression, and anxiety.

### 4.3 The PAG in headache and pain

The PAG plays an important role in the endogenous pain and emotional modulatory system that it can regulate incoming nociceptive information and other kinds of signals, including cognitive behaviors, emotional states, and stress reaction.^12^, ^33^, ^34^ According to anatomy, PAG was divided into 4 functional areas: (1) dorsomedial (dmPAG); (2) dorsolateral (dlPAG); (3) lateral (lPAG); (4) ventrolateral (vlPAG). The dmPAG, lPAG, and vlPAG can project downward to the brainstem, whereas the dlPAG does not. The vlPAG receives projections from the prefrontal cortices, insula, and central nucleus of the amygdala; innervates the parasympathetic nervous system; and is involved in emotions regulation, endogenous descending pain modulatory system, and innate fear.^35^, ^36^

As the largest gray matter nucleus in the diencephalon, the thalamus serves as the central hub for integrating and processing nociceptive signals.^12^ This study showed increased FC between the PAG and pulvinar medial thalamus. The PAG receives nociceptive input signals and projects them upward to the thalamus, which processes the information and projects it to the sensory cortex to participate in pain perception.^12^ Some evidence suggests that PAG can project substantial neurons to the paraventricular thalamic nucleus and is involved in processing acute and/or chronic stressors.^37^ Recent studies have also shown that PKC‐dependent signaling pathways in the PAG and thalamus are involved in pain processing,^38^ and reciprocal interactions in these regions may be important for pain perception.^39^

Numerous studies have shown that the cingulate gyrus exhibits specific regulatory functions in emotions, actions, and memory.^40^ The spatial and motor‐related information from the parietal cortex is projected to the posterior cingulate cortex and outputted from the midcingulate motor area to the premotor area. Additionally, the anterior cingulate cortex is involved in emotional processing and action, while the posterior cingulate cortex is involved in memory by outputting information to the hippocampus.^40^ Our study found a significant decrease in FC between the PAG and the cingulate cortex. We believe that the dysregulation of emotional, behavioral, and memory functions in the cingulate gyrus may be one of the pathological mechanisms of abnormal emotional behavior and memory in patients with NDPH. The posterior insula receives primary interoceptive signals, and it can process low‐level sensory features. This information is then relayed to the anterior insula and integrated emotional, cognitive, and motivational signals from cortical and subcortical areas, including the anterior cingulate cortex, amygdala, prefrontal cortex, etc. Human behavior involves the dynamic integration of emotions, cognition, and motivation, and its dysfunction is the pathological basis of many psychiatric disorders. Substantial studies have shown that dysfunction of the insula is the common pathological basis of many psychiatric diseases.^41^ Additionally, recent studies have shown that the insula is involved in auditory processing.^42^ Some studies have suggested that the superior temporal gyrus is involved in the perception of emotions in response to facial stimuli and auditory processing.^43^, ^44^ Our results illustrated significantly decreased FC between the PAG and insula and superior temporal gyrus. In summary, we believe the dysfunction of the insula and superior temporal gyrus may reveal the cause of the emotional disorder and phonophobia in patients with NDPH.

### 4.4 The reticular formation in headache and pain

The reticular formation, comprising a net‐like structure of brainstem nuclei and neurons, spans a wide area of the brainstem from the mesencephalon to the medulla oblongata and extends into the superior cervical spinal cord segments. It plays a pivotal role in coordinating a diverse range of reflexive and essential functions. These encompass arousal, consciousness, circadian rhythm regulation, sleep–wake cycles, somatic motor coordination, cardiovascular and respiratory control, pain modulation, as well as habituation.^45^, ^46^ Studies have shown that the reticular formation receives inputs from peripheral nociceptors and ascending pain pathways, enabling it to actively modulate incoming pain signals and activate the body's endogenous opioid system.^12^, ^46^, ^47^ This modulation can occur through inhibitory or facilitatory actions of the reticular formation on pain transmission, ultimately influencing the overall pain perception.^46^, ^48^ In the context of headaches, the reticular formation's connections with other regions implicated in headache pathophysiology suggest its contribution to headache generation.^46^ Our results showed that iMRt and mRt had decreased FC with cerebellum_9 and superior temporal gyrus, respectively, which indicated that the abnormal function of reticular formation may play a potential role in the pathogenesis of NDPH.

### 4.5 The parabrachial nucleus in headache and pain

The parabrachial nucleus, situated in the dorsolateral pons, encompasses the superior cerebellar peduncle as it enters the brainstem from the cerebellum. Anatomically, it comprises the medial parabrachial nucleus, lateral parabrachial nucleus, and subparabrachial nucleus. This nucleus plays a pivotal role in diverse physiological processes, including arousal, thermoregulation, and blood sugar regulation.^12^, ^49^, ^50^ Additionally, it is involved in pain modulation, including trigeminal pain.^51^ Furthermore, the rostral region of the parabrachial nucleus establishes connections with the rostral ventromedial medulla, facilitating access to the descending pain modulatory system.^52^ It also connects with higher subcortical structures such as the thalamus and hypothalamus for pain processing, as well as the amygdala for integration with the central autonomic network.^12^, ^49^ Our results showed that medial parabrachial nucleus had decreased FC with cerebellum_1 and cerebellum_2. There is substantial evidence that the cerebellum has extensive fibrous projection with parabrachial nucleus, and those projections involve in modulating classical fear conditioning.^53^, ^54^ However, future basic experiments are needed to verify the specific role of these related projections in headache, pain, and fear memories.

### 4.6 The ventral tegmental area in headache and pain

The ventral tegmental area (VTA), situated near the midline on the floor of the midbrain, serves as the source of dopaminergic cell bodies for the mesocorticolimbic dopamine system and other dopamine pathways. The VTA has been classified into four distinct zones or subareas by neuroscientists, namely the parabrachial pigmented area, paranigralis nucleus area, rostromedial tegmental nucleus, and parafasciculus retroflexus area. The paranigral and parabrachial areas are characterized by a dense population of dopamine neurons, while the parafasciculus and rostromedial regions exhibit a lower density of dopamine neurons. On the other hand, the paranigral and parabrachial area contains large‐sized and separately stained tyrosine hydroxylase neurons. The VTA plays a significant role in various processes, encompassing reward cognition, which involves motivational salience, associative learning, and positive emotions.^55^ Tamara demonstrated that reduced motivation induced by pain in rats is linked to decreased excitability and diminished activity of dopamine (DA) neurons in the VTA.^56^ Some evidence showed glutamatergic stimulation of VTA parabrachial pigmented nucleus neurons reduced nociceptive and spontaneous trigeminocervical complex neuronal firing.^57^

The cingulate cortex, positioned in the medial aspect of the cerebral cortex, constitutes a pivotal component of the limbic system, which is responsible for emotion formation and processing, as well as learning and memory.^58^ Given its crucial role, the cingulate cortex holds significant importance in disorders like depression and schizophrenia.^59^ Neurons from the VTA innervate the cingulate cortex. Our results showed that ventral tegmental area‐parabrachial pigmented nucleus complex had decreased FC with cingulate cortex. This may cause emotional and affective abnormalities in patients with NDPH. Additionally, we found decreased FC between the ventral tegmental area‐parabrachial pigmented nucleus complex and thalamus. The VTA receives glutamatergic afferents from the thalamus.^60^ These glutamatergic afferents play a critical role in modulating VTA cell firing. Activation of these neurons leads to an increase in firing rates of dopamine neurons within the VTA, inducing burst firing patterns.^60^ The cerebellum, with its extensive projections to the brainstem, is implicated in pain processing.^61^, ^62^ It receives pain input through descending cortico‐cerebellar pathways and ascending spino‐cerebellar pathways involving the pontine nuclei and inferior olives. Integration of this information within the cerebellum enables the transfer of relevant signals to the motor system, leading to a conscious motor response to pain that can be modulated according to pain intensity. Our results suggested that ventral tegmental area‐parabrachial pigmented nucleus complex had decreased FC with cerebellum. However, the pathophysiological mechanisms of cerebellar pain regulation in NDPH patients need to be further studied.

We found that FC values were significantly reduced in the VTA and substantia nigra of patients with NDPH. The dopaminergic neurons in the substantia nigra and ventral tegmental area of the midbrain project to different regions: the dorsolateral caudate/putamen and the ventromedial nucleus accumbens, respectively. These projections establish the mesostriatal and mesolimbic pathways. Disruptions in these pathways contribute to various disorders, including schizophrenia, Parkinson's disease, and attention deficit hyperactivity disorder.^63^ This suggests that patients with NDPH were more likely to have psychiatric comorbidities.

### 4.7 The laterodorsal tegmental nucleus in headache and pain

The laterodorsal tegmental nucleus (LDTg), a significant cluster of cholinergic cells situated in the periventricular mesopontine tegmentum, sends ascending cholinergic projections through relays in the rostral thalamus and basal forebrain. These projections have been traditionally associated with promoting cortical arousal and facilitating the onset of rapid eye movement sleep.^64^, ^65^ Moreover, LDTg are master regulators of the firing pattern of dopamine neurons, with tonic input from LDTg being imperative for glutamate elicited burst‐firing activity of VTA neurons.^66^, ^67^ Given these central functions, prior physiological and anatomical investigations of the LDTg have primarily emphasized its connections with the thalamus, basal forebrain, and VTA‐nigra complex. Previous studies have shown that voluntary wheel running in mice with neuropathic pain induces exercise‐induced hypoalgesia, potentially through the activation of the mesolimbic reward system mediated by LDTg and lateral hypothalamic area neurons.^68^ Positioned at the intersection of descending information from other brain areas and ascending nociceptive information from the spinal cord, the cerebellum is uniquely poised to modulate and be modulated by the processing of pain. However, our results showed decreased FC values between LDTg and cerebellum in patients with NDPH, which may suggest a novel underlying mechanism.

In conclusion, brain regions such as LC, raphe nucleus, PAG, reticular formation, parabrachial nucleus, VTA, and LDTg play important roles in NDPH. These regions constitute a complex neural circuit of headache, and abnormal FC may contribute to headache onset and exacerbation. For example, regions such as the LC and para‐brachial nucleus are involved in pain regulation and transmission, and when their FC is altered, it may lead to an exacerbation of pain perception. The raphe nucleus and reticular formation are involved in the regulation of vascular tone and blood flow. Abnormal activity of raphe nucleus and reticular formation may lead to vasodilation and blood flow changes, which may cause headache. In contrast, PAG, VTA, and LDTg are related to emotion regulation and stress, and their dysfunction may lead to worsening headache. Therefore, changes in FC of these brain regions are closely related to headache, and an in‐depth understanding of these neural mechanisms can help to better understand and treat headache symptoms.

### 4.8 Limitations

This study has certain limitations that should be acknowledged. Firstly, it is important to note that this study was conducted at a single center with a relatively small sample size, and thus, our preliminary findings require validation with larger datasets. Secondly, as a cross‐sectional study, we were unable to assess the changes in FC over the course of disease progression or determine their reversibility. Longitudinal studies are warranted to examine the correlation between FC and prognosis as well as disease progression. Moreover, the use of the 76 brainstem regions as seeds in this study limited our analysis to the whole regions without considering their subregions. Notably, different subregions of the seeds may possess distinct physiological functions that could influence FC analyses. Furthermore, it is crucial to recognize that psychiatric comorbidities, particularly chronic anxiety and depression, even if at a low grade and untreated, might impact FC in NDPH. Further investigations are necessary to assess whether such comorbidities contributed to the observed FC abnormalities. Therefore, future studies should explore the FC within the subregions of these seeds to enhance our understanding of the central pathogenesis of NDPH. Additionally, applying comparative analysis of established FC using cross‐correlation synchronization indices and dynamic FC between these different ROIs might yield significant conclusions. Conducting a subgroup analysis to explore differences between NDPH subgroups could uncover valuable insights, potentially differentiating NDPH more clearly from other headache types.

## 5 CONCLUSIONS

Given the persistent pain experienced in NDPH, the lack of identifiable causes, and the absence of evidence‐based treatment options, it is crucial to investigate this debilitating yet low‐incidence condition. This study aims to explore the FC of various brainstem regions in adults with NDPH. The results indicate abnormal FC in multiple brainstem nuclei implicated in pain and emotions processing and regulation among NDPH patients. Furthermore, these findings contribute to our understanding of central nervous system dysfunction underlying associated symptoms in NDPH and offer potential avenues for physicians to explore in the management of chronic pain.

## AUTHOR CONTRIBUTIONS

Wei Wang, Yonggang Wang, and Binbin Sui supported the conception and design of this project. Wei Wang, Dong Qiu, Yanliang Mei, Xiaoyan Bai, Ziyu Yuan, Xue Zhang, Zhonghua Xiong, Hefei Tang, Peng Zhang, Yaqing Zhang, and Xueying Yu acquired data. Wei Wang and Dong Qiu analyzed the data. Dong Qiu and Yanliang Mei contributed to data quality control. Wei Wang produced the first draft. All authors contributed intellectual content to the revised manuscript and have read and approved the final manuscript.

## CONFLICT OF INTEREST STATEMENT

The authors report no competing interests.

## TRIAL REGISTRATION

ClinicalTrials.gov Identifier: NCT05334927.

## Supporting information


Table S1.

## Data Availability

The data that support the findings of this study are available from the corresponding author upon reasonable request.
