# Supplementary material for: Altered functional connectivity of brainstem nuclei in new daily persistent headache: Evidence from resting‐state functional magnetic resonance imaging
Source: CNS Neurosci Ther. 2024 Mar 22;30(3):e14686. doi: 10.1111/cns.14686 (PMC10958407; doi:10.1111/cns.14686)
Supplement: Supplementary file 1 — Table S1. [file CNS-30-e14686-s001.docx]

**Table S1. Correlation between FC and clinical characteristics in patients with NDPH**

| Brain region | Side | BMI  *r* (*p*-value) | Disease duration  *r* (*p*-value) | VAS score  *r* (*p*-value) | HIT-6 score  *r* (*p*-value) | PHQ-9 score  *r* (*p*-value) | GAD-7 score  *r* (*p*-value) | PSQI score  *r* (*p*-value) | MoCA score  *r* (*p*-value) |
| --- | --- | --- | --- | --- | --- | --- | --- | --- | --- |
| Seed: iMRt_R;  FC: patients ＜ HCs |  |  |  |  |  |  |  |  |  |
| Cluster 1 |  |  |  |  |  |  |  |  |  |
| Cerebellum_9 | R | 0.003 (0.986) | -0.079 (0.063) | 0.293 (0.123) | 0.073 (0.727) | 0.167 (0.414) | 0.186 (0.364) | 0.267 (0.198) | -0.189 (0.453) |
| Seed: mRt_L;  FC: patients ＜ HCs |  |  |  |  |  |  |  |  |  |
| Cluster 1 |  |  |  |  |  |  |  |  |  |
| Temporal_Sup | R | -0.016 (0.936) | -0.285 (0.913) | 0.340 (0.071) | 0.253 (0.223) | -0.092 (0.655) | 0.211 (0.300) | -0.009 (0.967) | 0.074 (0.771) |
| Seed: LC_L;  FC: patients ＜ HCs |  |  |  |  |  |  |  |  |  |
| Cluster 1 |  |  |  |  |  |  |  |  |  |
| Cerebellum_Crus2 | L | 0.324 (0.087) | 0.114 (0.556) | -0.121 (0.530) | -0.124 (0.556) | -0.075 (0.714) | 0.149 (0.467) | -0.418 (0.149) | -0.038 (0.883) |
| Seed: LC_R;  FC: patients ＜ HCs |  |  |  |  |  |  |  |  |  |
| Cluster 1 |  |  |  |  |  |  |  |  |  |
| Cerebellum_Crus2 | L | 0.023 (0.908) | 0.143 (0.461) | 0.014 (0.943) | 0.010 (0.903) | -0.190 (0.352) | 0.075 (0.716) | 0.150 (0.413) | 0.191 (0.448) |
| Cerebellum_9 | R | 0.025 (0.891) | -0.002 (0.994) | 0.110 (0.570) | 0.003 (0.887) | -0.199 (0.330) | -0.050 (0.809) | 0.141 (0.502) | 0.220 (0.381) |
| Seed: LDTg_CGPn_L;  FC: patients ＜ HCs |  |  |  |  |  |  |  |  |  |
| Cluster 1 |  |  |  |  |  |  |  |  |  |
| Cerebellum_4_5 | L | 0.195 (0.311) | 0.017 (0.931) | -0.057 (0.771) | 0.167 (0.424) | -0.135 (0.512) | -0.034 (0.868) | -0.044 (0.836) | 0.110 (0.664) |
| Vermis_4_5 | R | 0.185 (0.335) | -0.123 (0.524) | -0.067 (0.731) | 0.120 (0.569) | -0.059 (0.775) | 0.021 (0.919) | 0.033 (0.875) | 0.122 (0.630) |
| Seed: LDTg_CGPn_R;  FC: patients ＜ HCs |  |  |  |  |  |  |  |  |  |
| Cluster 1 |  |  |  |  |  |  |  |  |  |
| Cerebellum_Crus1 | L | 0.199 (0.301) | 0.125 (0.519) | 0.062 (0.749) | 0.031 (0.885) | 0.141 (0.493) | 0.072 (0.726) | 0.063 (0.763) | 0.426 (0.078) |
| Cerebellum_6 | L | 0.262 (0.169) | -0.173 (0.369) | -0.111 (0.566) | 0.115 (0.583) | -0.326 (0.104) | -0.180 (0.378) | -0.064 (0.763) | 0.134 (0.596) |
| Cerebellum_Crus2 | L | 0.306 (0.106) | -0.344 (0.068) | -0.067 (0.729) | 0.036 (0.865) | -0.135 (0.511) | -0.115 (0.577) | -0.098 (0.640) | 0.093 (0.715) |
| Vermis_4_5 | L | 0.288 (0.129) | -0.029 (0.881) | -0.062 (0.748) | 0.139 (0.507) | -0.152 (0.460) | 0.076 (0.712) | -0.025 (0.905) | 0.095 (0.708) |
| Vermis_6 | L | 0.262 (0.169) | -0.173 (0.369) | -0.111 (0.566) | 0.115 (0.583) | -0.326 (0.104) | -0.180 (0.378) | -0.064 (0.763) | 0.134 (0.596) |
| Cerebellum_Crus2 | R | 0.319 (0.091) | -0.129 (0.506) | -0.205 (0.286) | -0.052 (0.804) | -0.275 (0.174) | -0.137 (0.503) | -0.175 (0.403) | 0.008 (0.975) |
| Seed: MnR;  FC: patients ＜ HCs |  |  |  |  |  |  |  |  |  |
| Cluster 1 |  |  |  |  |  |  |  |  |  |
| Cerebellum_Crus2 | L | 0.191 (0.321) | -0.118 (0.541) | 0.125 (0.519) | 0.158 (0.452) | -0.030 (0.137) | 0.240 (0.138) | -0.065 (0.759) | 0.058 (0.818) |
| Cluster 2 |  |  |  |  |  |  |  |  |  |
| Thal_VPL | L | 0.393 (0.035) | 0.025 (0.897) | -0.156 (0.421) | -0.045 (0.830) | -0.420 (0.033) | -0.075 (0.714) | -0.213 (0.307) | -0.125 (0.622) |
| Thal_VL | L | 0.319 (0.092) | -0.049 (0.799) | -0.005 (0.980) | -0.010 (0.961) | -0.130 (0.526) | 0.117 (0.568) | -0.158 (0.449) | -0.101 (0.690) |
| Cluster 3 |  |  |  |  |  |  |  |  |  |
| Precuneus | L | 0.085 (0.662) | 0.210 (0.275) | 0.111 (0.565) | 0.098 (0.643) | 0.068 (0.742) | 0.242 (0.234) | -0.065 (0.757) | -0.344 (0.162) |
| Cuneus | L | 0.087 (0.654) | 0.281 (0.139) | -0.103 (0.597) | -0.231 (0.657) | 0.025 (0.902) | 0.275 (0.173) | -0.127 (0.545) | -0.396 (0.104) |
| Cluster 4 |  |  |  |  |  |  |  |  |  |
| Cingulate_Mid | R | 0.176 (0.360) | -0.100 (0.606) | -0.071 (0.714) | 0.179 (0.392) | -0.176 (0.390) | -0.005 (0.982) | 0.127 (0.544) | -0.319 (0.197) |
| Cingulate_Mid | L | 0.200 (0.297) | -0.072 (0.702) | -0.209 (0.276) | 0.190 (0.363) | -0.227 (0.264) | -0.006 (0.976) | -0.195 (0.350) | -0.362 (0.140) |
| Seed: MPB_L;  FC: patients ＜ HCs |  |  |  |  |  |  |  |  |  |
| Cluster 1 |  |  |  |  |  |  |  |  |  |
| Cerebellum_Crus1 | L | 0.212 (0.271) | -0.089 (0.641) | -0.059 (0.761) | 0.139 (0.508) | -0.207 (0.311) | -0.163 (0.468) | -0.166 (0.581) | 0.249 (0.320) |
| Cerebellum_Crus2 | L | 0.246 (0.298) | -0.204 (0.985) | -0.018 (0.925) | 0.255 (0.219) | -0.029 (0.888) | -0.043 (0.833) | -0.057 (0.785) | 0.242 (0.332) |
| Seed: PAG;  FC: patients ＞ HCs |  |  |  |  |  |  |  |  |  |
| Cluster 1 |  |  |  |  |  |  |  |  |  |
| Thal_PuM | L | -0.092 (0.637) | 0.216 (0.259) | -0.334 (0.077) | 0.009 (0.966) | 0.045 (0.828) | 0.055 (0.789) | -0.052 (0.804) | 0.236 (0.346) |
| Thal_PuM | R | 0.007 (0.970) | 0.118 (0.543) | -0.043 (0.020) | -0.094 (0.650) | -0.062 (0.762) | 0.041 (0.844) | -0.140 (0.984) | 0.354 (0.150) |
| Seed: PAG;  FC: patients ＜ HCs |  |  |  |  |  |  |  |  |  |
| Cluster 1 |  |  |  |  |  |  |  |  |  |
| Temporal_Sup | R | 0.439 (0.017) | 0.035 (0.855) | -0.416 (0.025) | -0.431 (0.031) | -0.408 (0.039) | -0.311 (0.122) | -0.460 (0.021) | 0.067 (0.793) |
| Insula | R | 0.247 (0.197) | -0.152 (0.432) | -0.048 (0.028) | -0.604 (0.001) | -0.384 (0.084) | -0.280 (0.166) | -0.549 (0.004) | 0.280 (0.260) |
| Cluster 2 |  |  |  |  |  |  |  |  |  |
| Cingulate_Mid | L | 0.174 (0.367) | 0.211 (0.272) | -0.445 (0.016) | -0.131 (0.523) | -0.155 (0.451) | -0.018 (0.573) | -0.116 (0.448) | -0.445 (0.016) |
| Cingulate_Mid | R | 0.243 (0.204) | 0.124 (0.521) | -0.511 (0.005) | -0.240 (0.248) | 0.162 (0.430) | -0.209 (0.305) | -0.250 (0.227) | -0.058 (0.820) |
| Cluster 3 |  |  |  |  |  |  |  |  |  |
| Temporal_Sup | L | 0.251 (0.188) | 0.130 (0.501) | -0.340 (0.071) | -0.341 (0.095) | -0.205 (0.341) | -0.269 (0.184) | -0.285 (0.167) | 0.009 (0.605) |
| Insula | L | 0.093 (0.630) | -0.036 (0.854) | -0.458 (0.613) | -0.457 (0.822) | -0.249 (0.220) | -0.376 (0.116) | -0.418 (0.003) | 0.332 (0.118) |
| Seed: VTA_PBP_L;  FC: patients ＜ HCs |  |  |  |  |  |  |  |  |  |
| Cluster 1 |  |  |  |  |  |  |  |  |  |
| Cingulate_Mid | L | 0.100 (0.607) | -0.061 (0.753) | 0.239 (0.212) | 0.087 (0.680) | -0.184 (0.368) | -0.009 (0.964) | -0.045 (0.832) | -0.004 (0.985) |
| Thal_IL | R | 0.226 (0.239) | 0.088 (0.652) | 0.296 (0.119) | 0.157 (0.455) | 0.010 (0.981) | 0.153 (0.454) | 0.060 (0.776) | -0.153 (0.545) |
| Cluster 2 |  |  |  |  |  |  |  |  |  |
| Cerebellum_Crus1 | L | 0.342 (0.070) | -0.276 (0.148) | -0.120 (0.854) | -0.169 (0.418) | -0.159 (0.438) | -0.099 (0.630) | -0.223 (0.285) | 0.034 (0.894) |
| Cerebellum_Crus2 | L | 0.141 (0.467) | -0.371 (0.394) | -0.115 (0.551) | -0.189 (0.366) | -0.049 (0.812) | 0.022 (0.915) | -0.167 (0.925) | -0.021 (0.925) |
| Cerebellum_7b | L | 0.286 (0.133) | -0.289 (0.288) | -0.081 (0.677) | -0.074 (0.724) | -0.172 (0.402) | -0.020 (0.924) | -0.178 (0.354) | -0.121 (0.353) |
| Cluster 3 |  |  |  |  |  |  |  |  |  |
| Thal_VPL | R | 0.224 (0.243) | 0.136 (0.482) | 0.367 (0.051) | 0.178 (0.374) | 0.135 (0.512) | 0.195 (0.341) | 0.175 (0.404) | -0.100 (0.694) |
| Seed: VTA_PBP_R;  FC: patients ＜ HCs |  |  |  |  |  |  |  |  |  |
| Cluster 1 |  |  |  |  |  |  |  |  |  |
| Cerebellum_Crus1 | L | 0.203 (0.292) | -0.237 (0.216) | -0.265 (0.165) | 0.001 (0.974) | -0.222 (0.275) | -0.100 (0.626) | -0.001 (0.997) | 0.325 (0.188) |
| Cerebellum_Crus2 | L | 0.103 (0.594) | -0.390 (0.036) | -0.141 (0.466) | -0.127 (0.546) | -0.166 (0.418) | -0.049 (0.812) | -0.146 (0.487) | 0.097 (0.701) |
| Cluster 2 |  |  |  |  |  |  |  |  |  |
| SN | R | 0.507 (0.005) | -0.020 (0.916) | 0.274 (0.150) | 0.063 (0.765) | -0.129 (0.530) | 0.163 (0.427) | -0.055 (0.794) | -0.284 (0.253) |

Abbreviations: HCs, healthy controls; VAS, visual analogue scale; HIT-6, headache impact test; PHQ-9, patient health questionnaire-9; GAD-7, generalized anxiety disorder-7; PSQI, Pittsburgh sleep quality index; MoCA, Montreal cognitive assessment. FC, functional connectivity; HCs, healthy controls; MNI, Montreal Neurological Institute; L, left; R, right; FWE corr., family wise error correction; iMRt, inferior medullary reticular formation; LC, locus coeruleus; LDTg_CGPn, laterodorsal tegmental nucleus-central gray of the rhomboencephalon; MnR, median raphe; Thal_VPL, ventral posterolateral thalamus; Thal_VL, ventral lateral thalamus; Mid, middle; MPB, medial parabrachial nucleus; mRt, mesencephalic reticular formation; Sup, superior; PAG, periaqueductal gray; Thal_PuM, pulvinar medial thalamus; ACC, anterior cingulate cortex; PMnR, paramedian nucleus; RN, red nucleus; SN, substantia nigra; Thal_IL, intralaminar thalamus; VTA_PBP, ventral tegmental area-parabrachial pigmented nucleus complex.

Statistical significance: p < 0.00016 (Bonferroni correction).
